# Assessing the Metabolic and Physical Effects of Combined DPP4 and SGLT2 Inhibitor Therapy in Patients with Type-2 Diabetes Mellitus: An Observational Prospective Pilot Study

**DOI:** 10.31662/jmaj.2023-0214

**Published:** 2024-06-10

**Authors:** Ayako Nagayama, Tetsuaki Inokuchi, Kenji Ashida, Chizuko Inada, Tomoki Homma, Hiroshi Miyazaki, Takeki Adachi, Shimpei Iwata, Seiichi Motomura, Masatoshi Nomura, Kurume Medical Study Group of Internal Medicine

**Affiliations:** 1 Division of Endocrinology and Metabolism, Department of Internal Medicine, Kurume University School of Medicine, Kurume, Japan; 2 Inokuchi Medical Clinic, Kurume, Japan; 3 Inada Clinic of Internal Medicine, Kurume, Japan; 4 Homma Clinic of Cardiology and Internal Medicine, Kurume, Japan; 5 Miyazaki Clinic of Cardiology and Internal Medicine, Kurume, Japan; 6 Adachi Clinic, Kurume, Japan

**Keywords:** diabetes mellitus, dipeptidyl peptidase 4 inhibitors, hand strength, sarcopenia, sodium-glucose transporter 2, therapy

## Abstract

**Introduction:**

This study aimed to assess the efficacy of combined administration of dipeptidyl peptide-4 (DPP4) and sodium-glucose cotransporter-2 (SGLT2) inhibitors on metabolic disorders and their preferable and complementary effects.

**Methods:**

The effectiveness of a 24-week intervention on metabolic parameters (including glucose profile), physical functions (grip strength and calf circumference), and health-related quality of life (HR-QOL) was analyzed using the International Physical Activity Questionnaire and Geriatric Depression Scale 5. A total of 39 patients with type-2 diabetes mellitus (T2DM) treated with the combination of DPP4 and SGLT2 inhibitors were included in this multicenter pilot study.

**Results:**

Combination therapy significantly reduced the HbA1c level (median [interquartile range]) after 24 weeks (pretreatment: 7.7% [7.3-8.2] vs. posttreatment: 7.1% [6.6-7.9], *P* < 0.001). The grip strength significantly increased after 24 weeks (1.7 ± 2.7 kg, *P* < 0.001), while the mean calf circumference and body mass index significantly decreased. In particular, administration of the SGLT2 inhibitor significantly increased total physical activity in participants aged ≥65 years (*P* = 0.003), while psychological QOL did not significantly improve.

**Conclusions:**

Combination therapy with DPP4 and SGLT2 inhibitors decreased HbA1c levels and improved physical function in patients with T2DM. This study confirmed the effectiveness of combination therapy for metabolic disorders and suggested its beneficial and complementary effects. Therefore, advances in treatment plans to achieve further improvements in glucose profiles using DPP4 and SGLT2 inhibitors are recommended to enhance the QOL of patients with T2DM. Clinical trial number: University Hospital Medical Information Network Center: UMIN000045375

## Introduction

Age-related dysfunctions, particularly physical, psychological, and cognitive disorders, in patients with type-2 diabetes mellitus (T2DM) are considered a major challenge when treating geriatric patients ^(1)^. The prognosis of patients with T2DM depends on the management of the progression of these disorders. Moreover, dietary restrictions and exercise-based therapies have limitations in achieving sustainable improvements. Thus, diabetes-associated medication therapy is required to manage plasma glucose levels and maximize the time when glucose levels are within the desired range ^(2)^.

Dipeptidyl peptidase-4 (DPP4) and sodium-glucose cotransporter-2 (SGLT2) inhibitors are candidate drugs that can reduce variations in plasma glucose levels ^(2)^. Linagliptin, a DPP4 inhibitor (DPP4i), enhances physiological glucagon-like peptide-1 (GLP-1) activity. Furthermore, GLP-1 exhibits pancreatic action through α- and β-islet cells and extra-pancreatic action ^(3)^. DPP4i increase skeletal muscle mass volume and bone mineral density ^(4)^ and improve cognitive function ^(5)^.

Empagliflozin, an SGLT2 inhibitor (SGLT2i), exerts plasma glucose-lowering effects via increased urinary glucose excretion. Recent clinical trials have reported various beneficial effects of SGLT2i, such as risk reduction of cardiovascular events and renal failure, thus reducing the mortality rate associated with diabetes mellitus ^(6), (7)^. In addition, it has been found that long-term SGLT2i treatment causes beneficial changes in body composition related to improvements in insulin sensitivity and hyperglycemia ^(8)^. Moreover, SGLT2i treatment has also been found to improve grip strength ^(9)^.

Combination therapy with SGLT2i and DPP4i can improve hyperglycemia without negatively affecting adherence to the therapy ^(10)^. A prospective study with single use of SGLT2i or DPP4i noted the superiority of SGLT2i in body weight reduction and DPP4i in decreasing plasma glucose variability ^(11)^. However, it remains unclear whether combination therapy with SGLT2i and DPP4i further improves physical dysfunction and health-related quality of life (HR-QOL) or not. Therefore, this prospective pilot study aims to investigate improvements in physical, psychological, and metabolic conditions in patients with T2DM treated with the combination of SGLT2i and DPP4i. The findings of this study provide valuable insights into the effectiveness of combination therapy on metabolic disorders and its preferable and complementary effects.

## Materials and Methods

### Study design and patient recruitment

**Figure 1.**
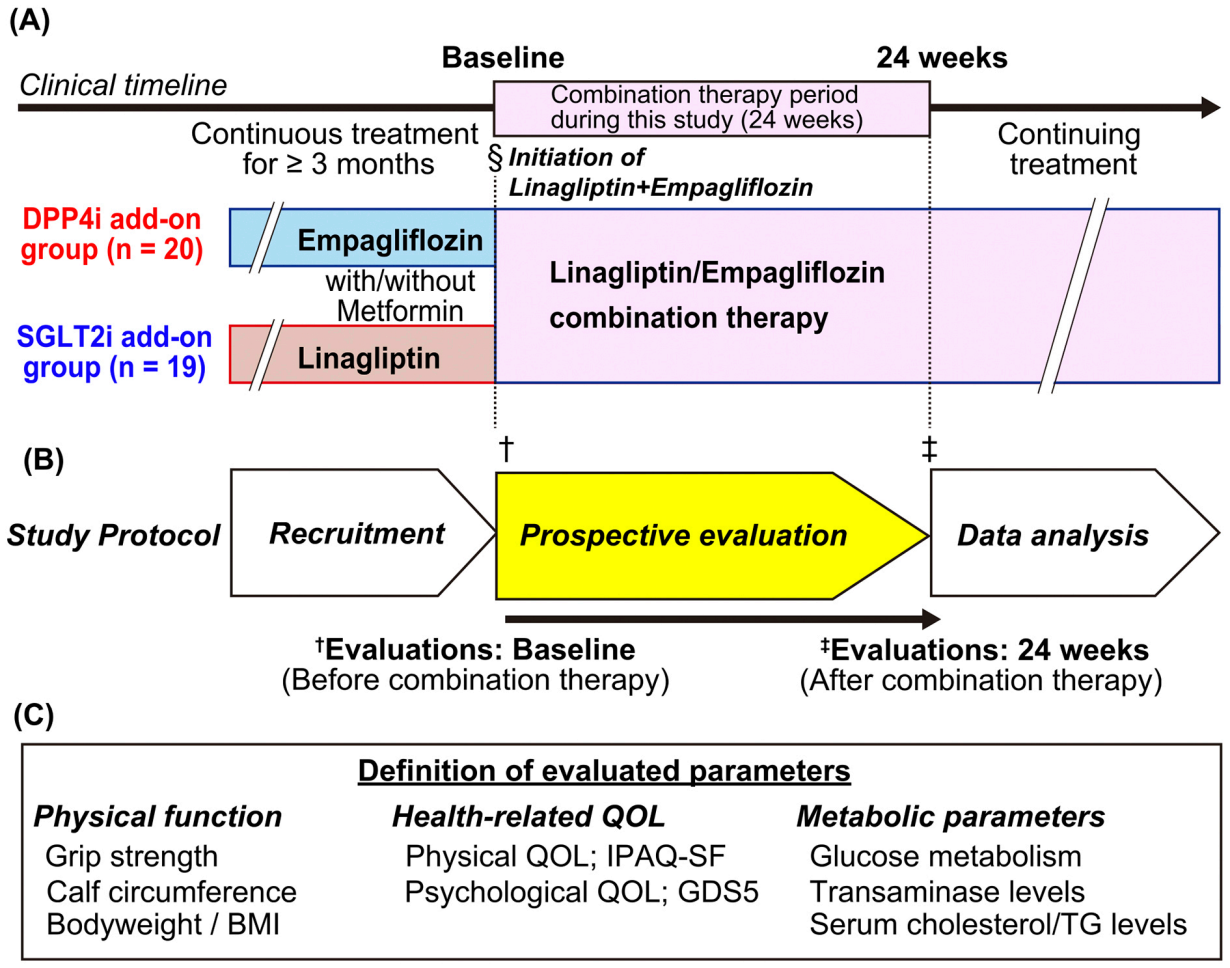
Study flowchart and definition of the evaluated parameters (A) Study flowchart: Patients with type-2 diabetes mellitus who had been treated with linagliptin or empagliflozin for ≥3 months were included in this study. The clinical data at both baseline and 24 weeks were evaluated. In addition, the patients were separated into two subgroups based on the preceding medications: the DPP4i and SGLT2i add-on groups. The clinical parameters were evaluated before (baseline) and after (24 weeks) combination therapy. (B) Timeline of the study protocol. (C) Definitions of parameters evaluated in this study are listed. BMI, body mass index; DPP4i, dipeptidyl peptidase-4 inhibitor; GDS5, Geriatric Depression Scale 5; IPAQ-SF, International Physical Activity Questionnaire Short Form; QOL, quality of life; SGLT2i, sodium-glucose cotransporter-2 inhibitor.

This open-label study included outpatients with T2DM treated at multiple medical institutes associated with Kurume University Hospital between September 5, 2019, and June 30, 2020. Patients aged >20 years with a glycated hemoglobin (HbA1c) level ≥7.0% (National Glycohemoglobin Standardization Program [NGSP]) who were treated with empagliflozin or linagliptin for ≥3 months were included. This study included all patients who met the inclusion criteria and who provided their informed consent. The participants were administered a combined tablet of empagliflozin and linagliptin instead of empagliflozin or linagliptin alone (Figure 1A). After a 24-week treatment of regular practical medical treatments, clinical parameters were evaluated in an observational study. Diet and exercise for the management of T2DM were uniformly provided in each institute. Patients with urinary tract infections, such as pyelonephritis, and impaired renal function (estimated glomerular filtration rate <30 mL/min/1.73 m^2^) were excluded. In addition, we divided patients using combination therapy with empagliflozin and linagliptin according to their prior medications (Figure 1A):

・DPP4i add-on group: patients who had been on empagliflozin for ≥3 months before combination therapy

・SGLT2i add-on group: patients who had been on linagliptin for ≥3 months before the combination therapy

This study was conducted in accordance with the ethical principles of the Declaration of Helsinki. The Ethics Committee of Kurume University School of Medicine approved this study (approval number: 19,034). Written informed consent was obtained from all participants. All procedures were performed following the ethical standards of the Institutional Review Board of Kurume University School of Medicine.

### Primary and secondary outcomes

The primary outcome of this study was a decrease in HbA1c levels by ≥0.5% (NGSP) measured at baseline and at 24 weeks after combination therapy, while the secondary outcomes were an increase in grip strength and improvements in physical and psychological QOL.

### Evaluation of physical functions

Body weight, blood pressure, and heart rate recorded at baseline and at 24 weeks were evaluated (Figure 1A and 1B). Hypertension was defined as systolic blood pressure ≥140 mmHg, diastolic blood pressure ≥90 mmHg, or antihypertensive drug use. The skeletal muscle volume in the legs was analyzed by measuring the circumference of the lower thighs, and a cutoff value of <31.0 cm was used to indicate sarcopenia ^(12), (13), (14)^. The bilateral handgrip strength in the standing position was analyzed, and the sarcopenia cutoff value in Japan was <26 kg and <18 kg for men and women, respectively ^(14), (15)^. Grip strength was measured four times for the right and left alterations using a Smedley-type hand dynamometer (Tsutsumi Seisakusho, Chiba, Japan) at each visit, and the maximum value among all measurements was used for the analysis.

### Evaluation of the HR-QOL

HR-QOL was assessed as physical and psychological QOL (Figure 1C). Physical QOL was evaluated using the International Physical Activity Questionnaire Short Form (IPAQ-SF). Specific activity types, such as walking and moderate-to-vigorous-intensity activities, were assessed following the guidelines for data processing and analysis of the International Physical Activity Questionnaire 2005 ^(16)^. Physical activity levels were converted to metabolic equivalent scores in accordance with the protocol ^(16)^. These scores were recorded at baseline and at 24 weeks during the study (Figure 1A and Figure 1B). Geriatric Depression Scale 5 (GDS5) was used to evaluate psychological QOL, which was analyzed at baseline and at 24 weeks.

### Criteria of diabetes-associated complications

・Retinopathy was defined as simple diabetic retinopathy or worse.

・Neuropathy included cardiac autonomic neuropathy and definitive clinical neuropathy. Sensory loss and ankle reflexes were graded as reduced and painful.

・Nephropathy included sustained microalbuminuria, which was defined as urinary albumin ≥30 mg/g·Cre at any two consecutive visits, and macroalbuminuria, which was defined as urinary albumin ≥300 mg/g·Cre at any visit.

### Measurements of metabolic parameters

Metabolic parameters, such as the levels of random plasma glucose, HbA1c, low-density lipoprotein (LDL) cholesterol, triglycerides, alanine aminotransferase (ALT), aspartate aminotransferase (AST), uric acid, and creatinine, were measured at each hospital visit according to standard procedures. The presence of glucose and protein in urine was also qualitatively determined.

### Stratified analysis

Stratified analysis was retrospectively performed according to the order of administration of SGLT2i and DPP4i, age (≥65 or <65 years), and prior administration of metformin (Figure 1A). The variations in physical functions, HR-QOL, and metabolic parameters between the DPP4i and SGLT2i add-on groups were analyzed.

### Statistical analysis

Power analysis was performed by setting a clinically meaningful decrease in HbA1c levels to 0.5% ^(17)^. The required number of participants, when the α and β error values were set to 0.05 and 0.2, respectively, was 17. Thus, it was confirmed that the number of study participants was sufficient to investigate the primary outcomes. All missing data were excluded from the analysis.

The Shapiro-Wilk test was used to assess the normality of the distribution of continuous data. Normally distributed data are expressed as the mean ± standard deviation (SD), while those with skewed distributions as median with interquartile range (IQR). Changes in physical functions, HR-QOL, and metabolic parameters before (baseline) and after (24 weeks) combination therapy were analyzed using the paired sample *t*-test or Wilcoxon signed-rank test. The comparison between the DPP4i and SGLT2i add-on groups regarding physical functions, HR-QOL, and metabolic parameters showing normal or nonnormal distribution was analyzed using Student’s *t*-test or Mann-Whitney *U* test, respectively. JMP Pro 17 software (SAS Institute Inc., NC, USA) was used to perform statistical analyses. A *P* value of <0.05 was considered statistically significant.

## Results

### Baseline characteristics

Table 1 shows the clinical characteristics of the 39 study participants (20 men and 19 women) at 24 weeks. The mean age was 64 ± 11 years, and 18 (46%) patients were aged ≥65 years. The mean duration since the diagnosis of T2DM was 12 ± 8 years. There were no significant differences between the DPP4i (n = 20) and SGLT2i add-on groups (n = 19) in sex, age, mean duration since T2DM diagnosis, diabetic complications, and medications used, except for DPP4i and SGLT2i use (Table 1). Regarding the evaluations at baseline, the median HbA1c value (NGSP) was 7.7% (IQR: 7.3-8.2, range: 6.6-10.9) (Table 2).

**Table 1.** Characteristics of the Participants in This Cohort Study.

Variable	Overall (n = 39)	DPP4i add-on group (n = 20)	SGLT2i add-on group (n = 19)	*P*
Number (male/female)	20/19	11/9	9/10	0.75^†^
Age (years)	64 ± 11	61 ± 12	67 ± 10	0.079^‡^
≥65 years of age, n (%)	18 (46%)	7 (35%)	11 (58%)	0.20^†^
<65 years of age, n (%)	21 (54%)	13 (65%)	8 (42%)	0.20^†^
Duration of diabetes (years)	12 ± 8	12 ± 7	12 ± 9	0.58^‡^
Diabetes-associated complications
Retinopathy	4	2	2	>0.99^†^
Neuropathy	8	4	4	>0.99^†^
Nephropathy	11	3	8	0.082^†^
Medications for diabetes mellitus
Metformin	24	15	9	0.10^†^
Sulfonylurea	10	6	4	>0.99^†^
Insulin	5	3	2	>0.99^†^
α-Glucosidase inhibitor	1	1	0	>0.99^†^
Grip strength (kg)	27.5 ± 9.6	30.3 ± 10.4	24.5 ± 8.3	0.058^‡^
Calf circumference (cm)
Right	36.9 ± 3.6 (n = 37)	36.9 ± 3.7 (n = 18)	36.9 ± 3.6 (n = 19)	0.98^‡^
Left	37.0 ± 3.1 (n = 37)	36.8 ± 3.3 (n = 18)	37.2 ± 3.1 (n = 19)	0.75^‡^
Adherence to medication score	7 (6-7)	7 (6-7)	7 (6-7)	0.98^§^
GDS5 (total score)	1 (0-3)	1 (0-2)	0 (0-3)	0.25^§^
IPAC-SF 2 (total physical activity score)	792 (240-2,919)	1,053 (478-4,345)	438 (66-2,772)	0.050^§^

Continuous variables that showed a normal or nonnormal distribution are expressed as mean ± SD or median (interquartile range (IQR)). Values were compared between the DPP4i and SGLT2i add-on groups using Fisher’s exact test (†), Student’s *t*-test (‡), or Mann-Whitney *U* test (§) DPP4i, dipeptidyl peptidase-4 inhibitor; GDS5, Geriatric Depression Scale 5; IPAC-SF, International Physical Activity Questionnaire Short Form 2; SGLT2i, sodium-glucose cotransporter-2 inhibitor

**Table 2.** Changes in Metabolic Parameters before and after Combination Therapy of DPP4 and SGLT2 Inhibitors.

Variable	Overall (n = 39)	DPP4i add-on group (n = 20)	SGLT2i add-on group (n = 19)	*P*
HbA1c^†^, %, median (IQR)				
Baseline	7.7 (7.3-8.2)	7.7 (7.2-8.5)	7.7 (7.5-8.3)	0.92^‡^
24 weeks	7.1 (6.6-7.9)	7.2 (6.6-7.9)	7.1 (6.5-7.7)	0.74^‡^
Change level	−0.70 (−1.2 to −0.3)	−0.65 (−0.9 to −0.4)	−0.70 (−1.2 to −0.3)	0.47^‡^
*p*‖	<0.001**	<0.001**	<0.001**
AST, IU/L, median (IQR)				
Baseline	20 (17-27) (n = 38)	20 (17-25) (n = 19)	19 (16-28) (n = 19)	0.71^‡^
24 weeks	19 (17-24) (n = 37)	19 (15-24) (n = 19)	19 (17-26) (n = 18)	0.71^‡^
Change level	−2 (−4 to 1) (n = 36)	−1 (−5 to 1) (n = 18)	−2 (−4 to 0) (n = 18)	0.43^‡^
*p*‖	0.046*	0.32	0.090
ALT, IU/L, median (IQR)				
Baseline	23 (16-35)	22 (16-27)	23 (16-36)	0.75^‡^
24 weeks	18 (14-28)	17 (13-28)	20 (14-26)	0.97^‡^
Change level	−3 (−9 to 1)	−3 (−7 to 2)	−6 (−9 to 0)	0.23^‡^
*p*‖	<0.001**	0.10*	0.003**
Triglyceride, mg/dL, median (IQR)				
Baseline	148 (110-231) (n = 39)	184 (110-293) (n = 20)	142 (127-197) (n = 19)	0.19^‡^
24 weeks	131 (97-206) (n = 37)	131 (97-309) (n = 19)	129 (91-165) (n = 18)	0.29^‡^
Change level	−19 (−76 to −18) (n = 37)	−19 (−94 to 5) (n = 19)	−11 (−63 to 26) (n = 18)	0.35^‡^
*p*‖	0.066	0.083	0.080
LDL cholesterol, mg/dL, mean ± SD				
Baseline	116 ± 28 (n = 39)	112 ± 30 (n = 20)	121 ± 26 (n = 19)	0.36^§^
24 weeks	114 ± 30 (n = 38)	112 ± 26 (n = 19)	117 ± 35 (n = 19)	0.56^§^
Change level	−3 ± 20 (n = 38)	−2 ± 17 (n = 19)	−3 ± 23 (n = 19)	0.89^§^
*p*^¶^	0.38	0.53	0.54

Continuous variables that showed a normal or nonnormal distribution are expressed as mean ± standard deviation (SD) or median (interquartile range (IQR)), respectively. †, the median hemoglobin A1c level is presented as NGSP. The difference in values between the DPP4i and SGLT2i add-on groups was analyzed using Mann-Whitney *U* test (‡) or Student’s *t*-test (§). Comparison of each value between baseline and 24 weeks was performed using Wilcoxon signed-rank test (‖) or the paired sample *t*-test (¶). *P < 0.05, ** *P* < 0.01 ALT, alanine transaminase; AST, aspartate transaminase; DPP4i, dipeptidyl peptidase-4 inhibitor; IQR, interquartile range; LDL, low-density lipoprotein; NGSP, National Glycohemoglobin Standardization Program; SGLT2i, sodium-glucose cotransporter-2 inhibitor

Twenty-three (59%) patients had hypertension. Ten (27%) and five (13%) participants had high levels of ALT (>30 IU/L) and AST (>30 IU/L), respectively. Moreover, 23 (59%) participants had dyslipidemia (LDL cholesterol level >120 mg/dL or serum triglyceride level >150 mg/dL). Furthermore, there was no significant difference in the mean HbA1c level at 24 weeks between these groups (Table 2). Overall, the median body mass index (BMI) was 25.6 kg/m^2^ (IQR: 23.6-28.4), and 23 patients (59%) were diagnosed with obesity higher than the class I standard according to the Japanese obesity disease criteria (Table 3) ^(18)^.

**Table 3.** Changes in Physical Parameters before and after Combination Therapy of DPP4 and SGLT2 Inhibitors.

Variable	Overall (n = 39)	DPP4i add-on group (n = 20)	SGLT2i add-on group (n = 19)	*P*
Body mass index, kg/m^2^, median (IQR)				
Baseline	25.6 (23.6-28.4)	26.1 (22.8-28.1)	25.3 (24.1-28.9)	0.84^†^
24 weeks	25.3 (23.1-28.1)	25.9 (22.5-28.1)	24.1 (23.2-27.8)	0.77^†^
Change level	−0.49 (−1.04 to 0.17)	0.00 (−0.60 to 0.34)	−0.94 (−1.41 to −0.37)	0.002^†,**^
*p*^§^	0.001**	0.78	<0.001**
Blood pressure, mmHg, median (IQR)				
Systolic	(n = 36)	(n = 36)	(n = 19)
Baseline	127 (120-134)	127 (120-134)	130 (120-136)	0.40^†^
24 weeks	128 (124-132)	130 (123-134)	128 (124-132)	0.35^†^
Change level	0 (−6 to 5.75)	−4 (−8 to 6)	0 (−3 to 3.5)	0.18^†^
*p*^§^	0.78	0.28	0.25
Diastolic	(n = 36)	(n = 17)	(n = 19)
Baseline	68 (60-76)	66 (60-76)	70 (62-78)	0.34^†^
24 weeks	70 (60-74)	70 (61-74)	66 (60-74)	0.69^†^
Change level	0 (−6 to 4)	0 (−3 to 8)	−2 (−8 to 4)	0.21^†^
*p*^§^	0.57	0.59	0.25
Grip strength, kg, mean ± SD				
Baseline	27.5 ± 9.6	30.3 ± 10.4	24.5 ± 8.3	0.058^‡^
24 weeks	29.3 ± 9.8	31.7 ± 10.5	26.7 ± 8.6	0.11^‡^
Change level	1.7 ± 2.7	1.3 ± 2.2	2.1 ± 3.0	0.36^‡^
*p*‖	<0.001**	0.014*	<0.001**
Calf circumference, cm, mean ± SD				
Right	(n = 37)	(n = 18)	(n = 19)
Baseline	36.9 ± 3.6	36.9 ± 3.7	36.9 ± 3.6	0.98^‡^
24 weeks	36.4 ± 2.8	36.7 ± 2.9	36.1 ± 2.8	0.34^‡^
Change level	−0.66 ± 1.85	−0.22 ± 2.16	−1.05 ± 1.47	0.19^‡^
*p*‖	0.12	0.67	0.080
Left	(n = 37)	(n = 18)	(n = 19)
Baseline	37.0 ± 3.1	36.8 ± 3.3	37.2 ± 3.1	0.75^‡^
24 weeks	36.3 ± 2.9	36.7 ± 2.9	36.0 ± 3.0	0.65^‡^
Change level	−0.55 ± 1.42	−0.06 ± 1.36	−0.94 ± 1.38	0.070^‡^
*p*‖	0.013*	0.67	0.006**

Continuous variables that showed a normal or nonnormal distribution are expressed as mean ± standard deviation (SD) or median (interquartile range (IQR)), respectively. The difference in values between the DPP4i and SGLT2i add-on groups was analyzed using Mann-Whitney *U* test (†) or Student’s *t*-test (‡). Comparison of each value between baseline and 24 weeks was performed using Wilcoxon signed-rank test (§) or the paired sample *t*-test (‖). *P < 0.05, ** *P* < 0.01 ALT, alanine transaminase; AST, aspartate transaminase; DPP4i, dipeptidyl peptidase-4 inhibitor; IQR, interquartile range; LDL, low-density lipoprotein; NGSP, National Glycohemoglobin Standardization Program; SGLT2i, sodium-glucose cotransporter-2 inhibitor

### Changes in metabolic parameters and physical functions induced by linagliptin or empagliflozin administration

Regarding the primary endpoint of this study, the mean HbA1c level significantly decreased by 0.70% (NGSP) after a 24-week combination therapy with DPP4 and SGLT2 inhibitors (*P* < 0.001). In addition, BMI and transaminase levels in the overall study cohort significantly decreased after a 24-week combination therapy (Table 2 and 3). Moreover, the grip strength significantly increased at 24 weeks compared with that at baseline: 1.7 ± 2.7 kg (*P* < 0.001) (Table 3). Furthermore, the calf circumference of the left side significantly decreased at 24 weeks compared with that at baseline (*P* = 0.013) (Table 3). However, there were no significant changes in systolic and diastolic blood pressures, serum triglyceride levels, and LDL cholesterol levels (Table 2 and 3).

In the DPP4i add-on group, the HbA1c level significantly decreased after 24-week linagliptin administration compared with that at baseline (*P* < 0.001) (Table 2), whereas the BMI did not significantly change compared with that at baseline (Table 3). There were no significant changes in blood pressure and transaminase, LDL cholesterol, and triglyceride levels between baseline and 24 weeks (Table 2 and 3). The grip strength significantly increased (*P* = 0.014) after the combination therapy, although there was no significant difference in calf circumference on either side during the 24-week intervention period (Table 3).

In the SGLT2i add-on group, HbA1c level (*P* < 0.001) and BMI (*P* < 0.001) significantly decreased after 24-week combination therapy (Table 2 and 3). However, there was no significant change in blood pressure, transaminase, LDL cholesterol, and triglyceride levels. Furthermore, grip strength significantly increased after 24-week combination therapy (*P* < 0.001), whereas calf circumference on the left side significantly decreased (*P* = 0.006) (Table 3).

### Effect of empagliflozin or linagliptin administration on physical and psychological QOL

There were no significant changes in the median values of both total activity scores using the IPAQ-SF and GDS5 after 24-week combination therapy with SGLT2 and DPP4 inhibitors in patients with T2DM (Table 4). High adherence to medication was noted in this study.

**Table 4.** Changes in Health-Related QOL Evaluation Parameters before and after Empagliflozin and Linagliptin Combination Therapy.

Variable	Overall (n = 39)	DPP4i add-on group (n = 20)	SGLT2i add-on group (n = 19)	*P*
Adherence to medication score^†^				
Baseline	7 (6-7)	7 (6-7)	7 (6-7)	0.98^‡^
24 weeks	7 (6-7)	7 (6-7)	7 (6-7)	0.93^‡^
Change level	0 (0-0)	0 (0-0)	0 (0-0)	0.64^‡^
*p*^§^	0.72	>0.99	>0.99
Geriatric Depression Scale 5, total score
Baseline	1 (0-3)	1 (0-2)	0 (0-3)	0.25^‡^
24 weeks	1 (0-2)	1 (0-2)	2 (1-3)	0.12^‡^
Change level	0 (−0.75 to 0)	0 (−0.5 to 0)	0 (−1 to 0)	0.91^‡^
*p*^§^	0.32	0.35	0.50
International Physical Activity Questionnaire Short Form 2, total physical activity score				
Baseline	792 (240-2,919)	1,053 (478-4,345)	438 (66-2,772)	0.050^‡^
24 weeks	720 (240-2,200)	1,369 (320-3,308)	540 (135-1,260)	0.16^‡^
Change level	0 (−300-342)	21 (−573-722.5)	0 (−172-310)	>0.99^‡^
*p*^§^	0.59	0.76	0.57

Continuous variables are expressed as median (interquartile range (IQR)). †, adherence-medication score was calculated by analyzing questionnaires about medication from the participants. The difference in values between the DPP4i and SGLT2i add-on groups was analyzed using Mann-Whitney *U* test (‡). Comparison of each value between baseline and 24 weeks was performed using Wilcoxon signed-rank test (§). DPP4i, dipeptidyl peptidase-4 inhibitor; SGLT2i, sodium-glucose cotransporter-2 inhibitor

### Comparison of the changes in metabolic, physical, and psychological parameters between the DPP4i and SGLT2i add-on groups: a stratified analysis

**Figure 2.**
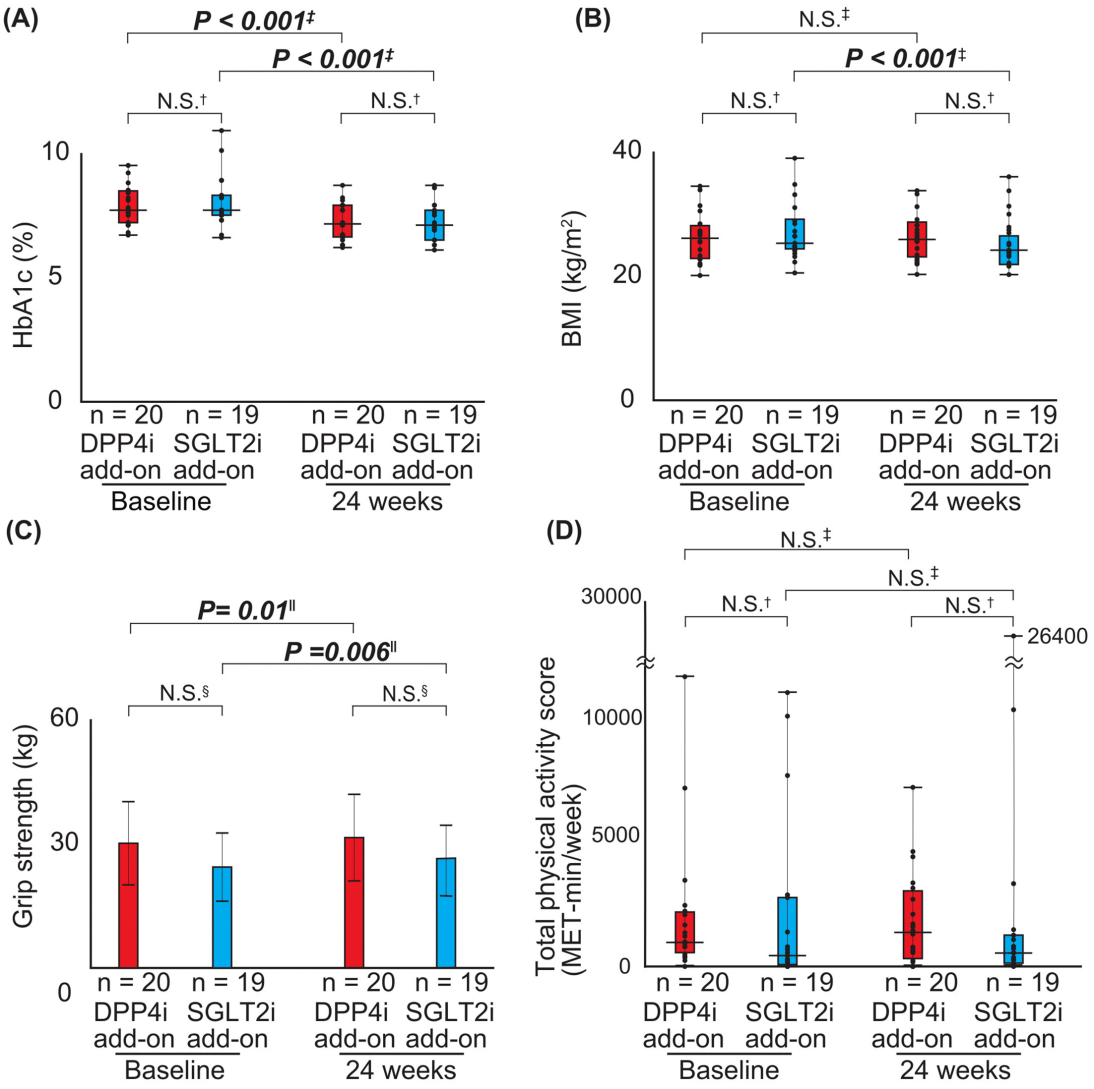
Comparison of the change values in parameters between baseline and 24 weeks (A) Hemoglobin A1c (HbA1c) levels, (B) body mass index (BMI), (C) grip strength, and (D) total physical activity scores compared to baseline and follow-up in DPP4i (n = 20; red box) and SGLT2i (n = 19; blue box) add-on groups. (A, B, D) Analyses were performed using Mann-Whitney *U* test (†) or Wilcoxon signed-rank test (‡). (C) Analyses were performed using Student’s *t*-test (§) or paired *t*-test (‖). BMI, body mass index; DPP4i, dipeptidyl peptidase-4 inhibitor; HbA1c, hemoglobin A1c; SGLT2i, sodium-glucose cotransporter-2 inhibitor.

Table 2, 3, and 4 and Figure 2 show the differences in the change in values for each parameter between the groups. There were no significant differences in the mean HbA1c levels between the DPP4i and SGLT2i add-on groups after 24-week combination therapy (Table 2 and Figure 2). Thus, the parameters were retrospectively evaluated based on the levels at 24 weeks when all participants were treated with both DPP4 and SGLT2 inhibitors. HbA1c levels significantly decreased in both the DPP4i (*P* < 0.001) and SGLT2i add-on groups (*P* < 0.001) (Table 2 and Figure 2A). In addition, ALT levels decreased in the SGLT2i add-on group (*P* = 0.003), but not in the DPP4i add-on group (Table 2). Body weight and BMI significantly decreased in the SGLT2i add-on group than in the DPP4i add-on group (*P* = 0.002) (Table 3 and Figure 2B). Although grip strength significantly increased after combination therapy (DPP4i add-on group: *P* = 0.014) (SGLT2i add-on group: *P* < 0.001), there were still no significant differences between the groups (*P* = 0.36) (Table 3 and Figure 2C). The calf circumference of the left side significantly decreased after combination therapy in overall, with a greater decrease in the SGLT2i add-on group (Table 3). There was no significant difference in health-related QOL (adherence to medication score, GDS5, and IPAQ-SF) between the groups during the 24-week combination therapy (Table 4 and Figure 2D).

**Figure 3.**
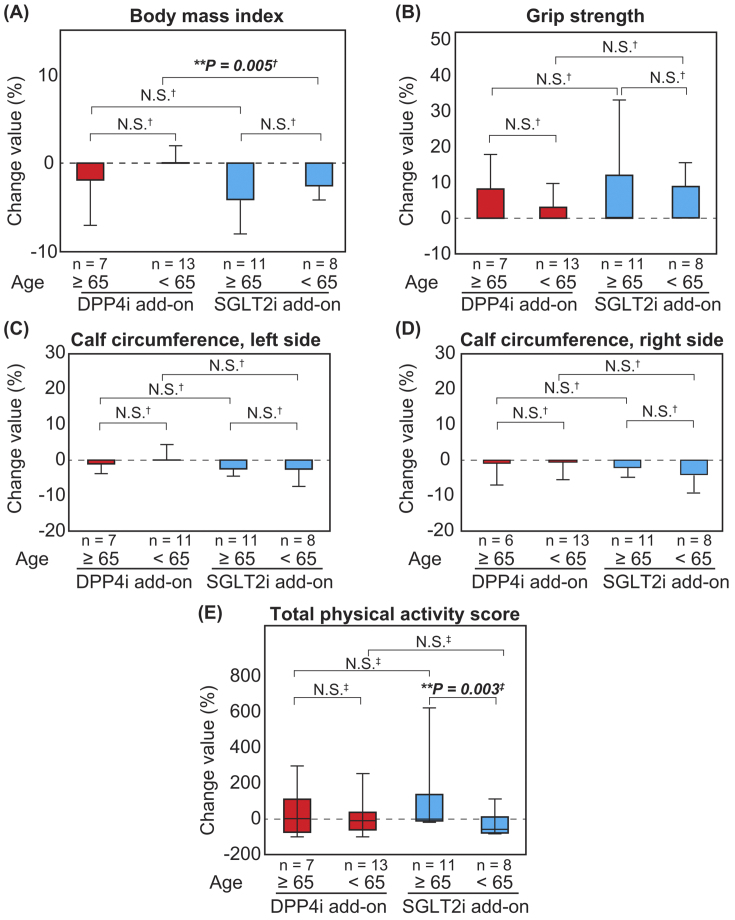
Changes in physical functions and QOL in the DPP4i (n = 20) and SGLT2i (n = 19) add-on groups Changes in the body mass index, grip strength, calf circumference, and total physical activity score of the SGLT2i and DPP4i add-on groups are presented. Each value is presented as the mean (red box, DPP4i add-on group; blue box, SGLT2i add-on group) and standard deviation (bars) for BMI, grip strength, and calf circumference or median (horizontal lines in colored boxes), interquartile range (IQR) (colored boxes), and range (bars) for total physical activity score. (A) Body mass index (BMI). (B) Grip strength. (C) Calf circumference, left. (D) Calf circumference, right. (E) Total physical activity score. Analyses were performed using Student’s *t*-test (†) or Mann-Whitney *U* test (‡). *P < 0.05, **P < 0.01. DPP4i, dipeptidyl peptidase-4 inhibitor; SGLT2i, sodium-glucose cotransporter-2 inhibitor.

**Figure 4.**
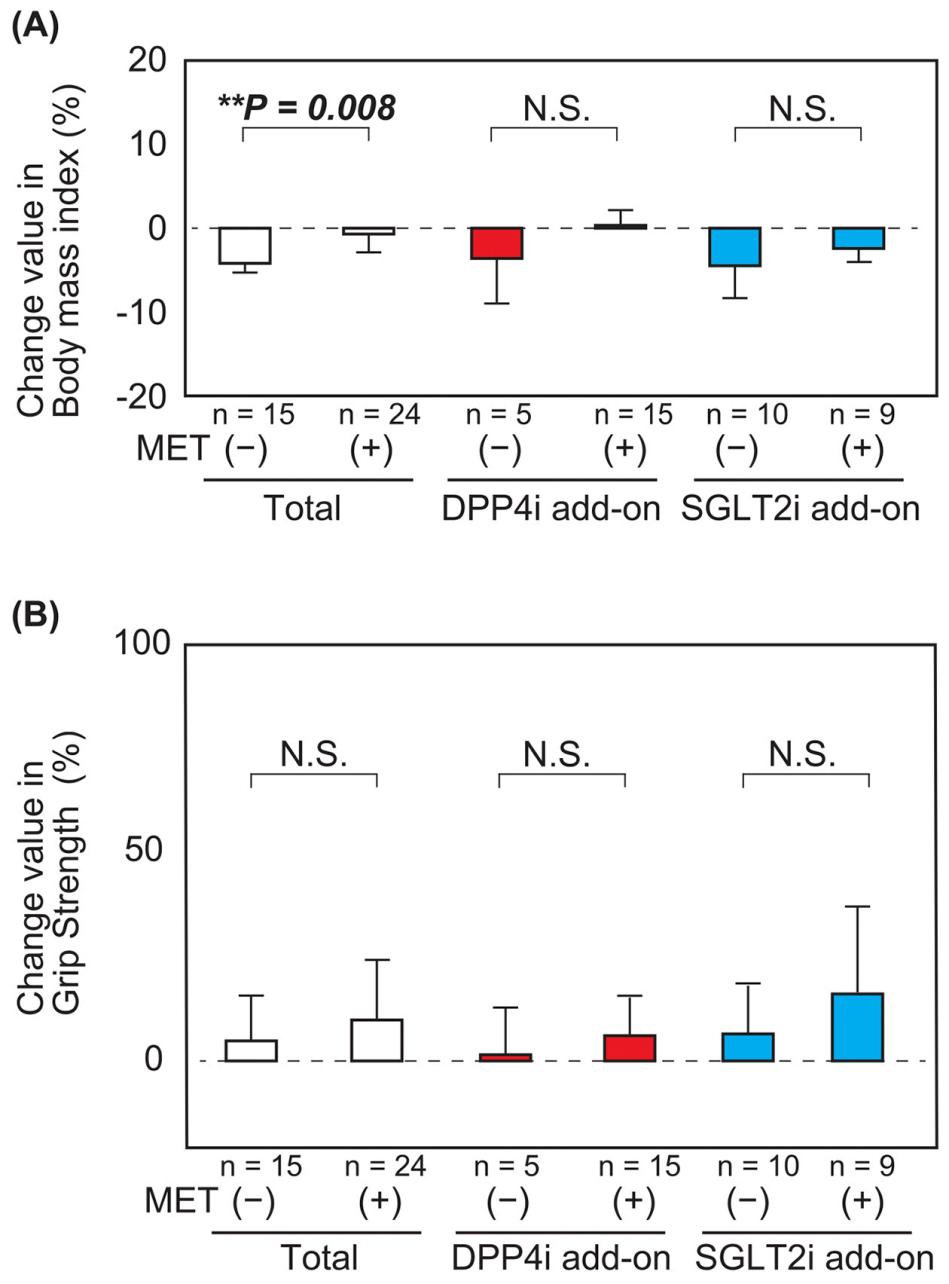
Changes in body weight and grip strength between before and after combination therapy with (n = 24) or without (n = 15) metformin preadministration (A) Stratified analysis based on DPP4i or SGLT2i add-on group showed significant differences between the groups with or without metformin preuse. Body weight significantly decreased after combination therapy with DPP4i and SGLT2i in the absence of metformin. (B) There was no significant difference in grip strength between the groups with or without metformin preuse. Values are expressed as mean (open box, total group; red box, DPP4i add-on group; blue box, SGLT2i add-on group) ± SD (bars). The total body mass index (without metformin: −4.19% ± 1.10% vs. with metformin: −0.70% ± 2.18%, *P* = 0.008). The DPP4i add-on group (without metformin: −3.61% ± 5.37% vs. with metformin: 0.34% ± 1.82%, *P* = 0.18). The SGLT2i add-on group (without metformin: −4.48% ± 3.86% vs. with metformin: −2.42% ± 1.61%, *P* = 0.15). Analyses were performed using Student’s *t*-test. *P < 0.05, **P < 0.01. DPP4i, dipeptidyl peptidase-4 inhibitor; SGLT2i, sodium-glucose cotransporter-2 inhibitor.

The BMI decreased in patients aged ≥65 years in both the DPP4i and SGLT2i add-on groups, although there was no significant difference (Figure 3A). There were no significant differences in the changes in the percentage values of grip strength and calf circumference after combination therapy between patients aged ≥65 years and <65 years (Figure 3B, 3C and 3D). Furthermore, there was no significant difference in the total physical activity score among patients in the DPP4i add-on group; however, there was a significant increase in the total physical activity score among patients aged ≥65 years than those aged <65 years in the SGLT2i add-on group (Figure 3E). With respect to the combination therapy with metformin and DPP4i or SGLT2i, the BMI of patients not on metformin was reduced to a greater extent than that of those on metformin (Figure 4A and 4B). This tendency was preserved in both DPP4i and SGLT2i add-on groups. In addition, there was no significant difference in the change in grip strength in the SGLT2i add-on group with metformin preuse than that without metformin preuse. Furthermore, there was no significant difference in the change in grip strength in the DPP4 add-on group, even when a stratified analysis based on metformin preuse was performed. There was also no significant difference in coadministration of sulfonylurea (Supplementary Figure 1) and insulin (Supplementary Figure 2) in the grip strength and calf circumferences.

## Discussion

This pilot study was conducted to confirm the safety and effectiveness of the combined DPP4 and SGLT2 inhibitors to improve hyperglycemia in patients with T2DM. This study demonstrated that the combination therapy may increase grip strength and reduce fat accumulation and improve hyperglycemia. Our study provides novel insights into setting effective therapeutic goals for patients with sarcopenic T2DM, although there were no significant improvements in physical and psychological QOL within 24 weeks of treatment.

This pilot study was conducted without any reports of adverse events. Combination therapy with DPP4 and SGLT2 inhibitors has been suggested as an effective therapy that maintains additive efficacy in improving hyperglycemia while retaining the safety profile of each medication. In addition, beneficial changes in anthropometric and physical parameters should be confirmed in future studies. More effective methods are required for future assessments of the physical and psychological QOL in patients with T2DM.

Additional administration of DPP4 or SGLT2 inhibitors to patients with T2DM effectively improved metabolic disorders. The addition of each drug significantly decreased the HbA1c levels of the participants. SGLT2 inhibitors reduce body weight and plasma glucose levels by enhancing urinary glucose excretion ^(8)^. Furthermore, SGLT2 inhibitors increase glucagon levels but not serum insulin levels, indicating that they act as metabolic regulators by enhancing lipolysis, resulting in free fatty acid and ketone production ^(19)^. SGLT2 inhibitors reportedly reduce HbA1c levels in patients with T2DM by an average of 1.0% ^(20)^ in an additive manner with DPP4 inhibitors ^(21), (22)^. Although SGLT2 inhibitors ameliorate hepatosteatosis ^(23)^, the role of DPP4 inhibitors remains controversial ^(24)^. The reduction in transaminase levels observed in our SGLT2 add-on group may demonstrate the difference between the effects of SGLT2 and DPP4 inhibitors in improving liver dysfunction. Furthermore, DPP4 inhibitors enhance insulin secretion by pancreatic β cells in a dose-dependent manner ^(25)^. The degree of reduction in HbA1c levels in the DPP4i add-on group was the same as that in a previous large cohort study ^(26)^.

The additional administration of DPP4 or SGLT2 inhibitors significantly reduced HbA1c levels, confirming the good compliance of the patients for their treatment. Furthermore, both medications increased grip strength, reduced fat accumulation, and improved hyperglycemia. The grip strength increased in an additive manner during the 24-week study period. The association between SGLT2i administration and changes in muscle volume and strength remains controversial. Clinicians should be aware of the risks of accelerating sarcopenia ^(27)^; however, an animal model study has suggested the preventive effects of obesity-related sarcopenia ^(28)^. In particular, SGLT2 inhibitors had a higher tendency to increase grip strength than DPP4 inhibitors. SGLT2 inhibitors significantly decreased the lower thigh circumference at 24 weeks than that at baseline; however, there was no significant difference between the groups. A lower thigh circumference of less than 31 cm indicates sarcopenia ^(29)^. SGLT2 inhibitors reportedly improve mitochondrial fatty acid oxidation in the skeletal muscle ^(30)^ and reduce body fat accumulation ^(31)^. This study suggests that the anti-metabolic actions of SGLT2 inhibitors are caused by the reduction in ectopic adipose accumulation. For instance, improvement in glucose intolerance is associated with reduced triglyceride content in the skeletal muscle ^(32)^ and steatosis in fatty liver disease ^(33)^. DPP4 inhibitors increase skeletal muscle volume by enhancing insulin secretion-related anabolic effects ^(34)^. Moreover, administration of DPP4 inhibitors increased the expression of glucose transporter type 4 ^(35)^ and improved mitochondrial function ^(36)^ in the muscles. Both SGLT2 ^(28)^ and DPP4 ^(37)^ inhibitors activate AMP kinase, leading to an increase in the muscle volume and strength. A stratified investigation, which indicated that prior metformin administration interfered with the effects of DPP4 and SGLT2 inhibitors, suggested that the mechanism of action of DPP4 and SGLT2 inhibitors shares pathways with that of metformin ^(38)^. Thus, treatment with DPP4 and SGLT2 inhibitors improves body composition, reduces fat accumulation, and increases skeletal muscle strength in patients with T2DM in an additive manner.

It has been reported that SGLT2 inhibitors maintain motivation for treatment and increase treatment satisfaction in patients with T2DM ^(39)^, while improvements in the plasma glucose profile also improve well-being in T2DM patients with hyperglycemia or insulin resistance ^(40)^. Diet intake ^(41)^, plasma glucose levels ^(42)^, ketones ^(43)^, insulin ^(44)^, incretins ^(45)^, and myokines ^(46)^ are possible factors responsible for ameliorating HR-QOL and physical activity levels in patients with T2DM. However, this study did not significantly improve physical and psychological QOL, although further studies over longer periods may confirm its effectiveness. SGLT2 inhibitors have been considered as key medications considering the order of drug initiation ^(22)^. The participants in the DPP4i add-on group, who were administered an SGLT2 inhibitor first, showed less adipose accumulation and more muscle quantity and quality than those in the SGLT2i add-on group, although there were no significant differences in the BMI and HbA1c levels. Although we could not draw firm conclusions, our results indicate that SGLT2 inhibitors may be primary candidate medications, as recommended for T2DM patients with congestive heart failure ^(47)^. Left side dominant decrease in thigh circumference may reflect the reductions in interstitial fluids depending on the anatomical features ^(48)^. Furthermore, SGLT2 inhibitors improve pancreatic β-cell responses to incretin hormones ^(49)^, and their preferential administration may affect the regulation of metabolic disorders, prevent complications, and preserve QOL and activities of daily living in patients with T2DM. Thus, clinicians should consider the order of these treatments.

This study had some limitations. First, this pilot study cannot exclude potential for bias, impact on accuracy, and uncertainties. Therefore, further large-scale prospective studies are processing to confirm and generalize our conclusions. Second, the IPAQ-SF may fail to determine the positive results behind the influences of social activities or seasonal variations, because it is a simplified questionnaire to investigate the physical activities of adults. Third, the GDS5 may not show significant changes due to the short period and not the older participants in this study.

### Conclusion

We performed a pilot study of the combination therapy using DPP4 and SGLT2 inhibitors for the future study. We investigated muscle quality and quantity by measuring grip strength and calf circumference, respectively, along with the HbA1c levels. Treatment with DPP4 and SGLT2 inhibitors improved hyperglycemia and grip strength and reduced fat accumulation. Thus, combination therapy with DPP4 and SGLT2 inhibitors demonstrated effectiveness and safety in patients with T2DM, although there were no improvements in physical and psychological QOL. Our study would provide basis for novel insights into the setting of effective therapeutic goals for patients with sarcopenic T2DM.

## Article Information

### Conflicts of Interest

None

### Acknowledgement

We thank all members of the committee of Internal Medicine in Kurume City, which consists of members from the Kurume University School of Medicine and local clinics, for their contribution to data collection and accomplishing this study. We also thank Editage (www.editage.com) for their English language editing services.

### Author Contributions

All authors contributed to the conception and design of the study. Ayako Nagayama and Tetsuaki Inokuchi contributed equally to this study. Ayako Nagayama, Tetsuaki Inokuchi, Kenji Ashida, Chizuko Inada, Tomoki Homma, Hiroshi Miyazaki, Takeki Adachi, Shimpei Iwata, Seiichi Motomura, and Masatoshi Nomura prepared the materials and collected and analyzed the data. Ayako Nagayama, Tetsuaki Inokuchi, Kenji Ashida, and Masatoshi Nomura wrote the first draft of the manuscript. All authors have read and approved the final manuscript.

### Approval by Institutional Review Board (IRB)

All procedures were performed in accordance with the ethical standards of the Institutional Review Board of Kurume University School of Medicine and conformed to the principles of the Declaration of Helsinki 2013. The Ethics Committee of Kurume University School of Medicine approved this study on June 10, 2019 (approval number: 19034).

### Informed Consent

Written informed consent was obtained from all participants. All procedures were performed following the ethical standards of the Institutional Review Board of Kurume University School of Medicine.

## Supplement

Supplementary Figure 1 Changes in grip strength and calf circumferences between patients with or without sulfonylurea administration
Analyses were performed using Student’s *t*-test.
DPP4i, dipeptidyl peptidase-4 inhibitor; SGLT2i, sodium-glucose cotransporter-2 inhibitor; SU, sulfonylurea

Supplementary Figure 2 Changes in grip strength and calf circumferences between patients with or without insulin administration
Analyses were performed using Student’s *t*-test.
DPP4i, dipeptidyl peptidase-4 inhibitor; SGLT2i, sodium-glucose cotransporter-2 inhibitor
