# Supplementary figures and images for: Assessing the Metabolic and Physical Effects of Combined DPP4 and SGLT2 Inhibitor Therapy in Patients with Type-2 Diabetes Mellitus: An Observational Prospective Pilot Study
Source: JMA J. 2024 Jun 10;7(3):387–400. doi: 10.31662/jmaj.2023-0214 (PMC11301034; doi:10.31662/jmaj.2023-0214)

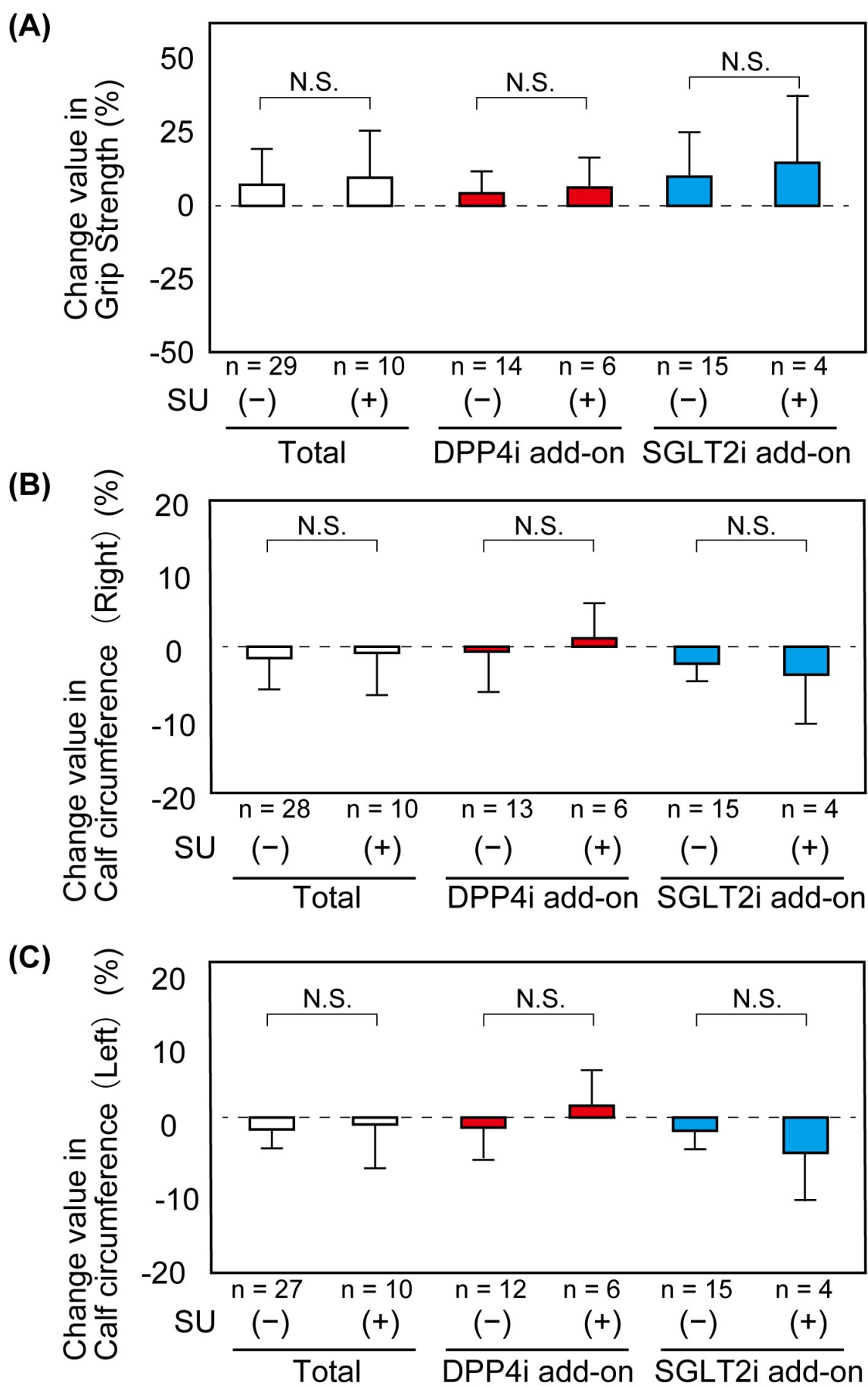

**Supplementary figure 1**

Supplement: Supplementary Figure 1 — Changes in grip strength and calf circumferences between patients with or without sulfonylurea administration Analyses were performed using Student’s t-test. DPP4i, dipeptidyl peptidase-4 inhibitor; SGLT2i, sodium-glucose cotransporter-2 inhibitor; SU, sulfonylurea [file 2433-3298-7-3-0387-s001.pdf]

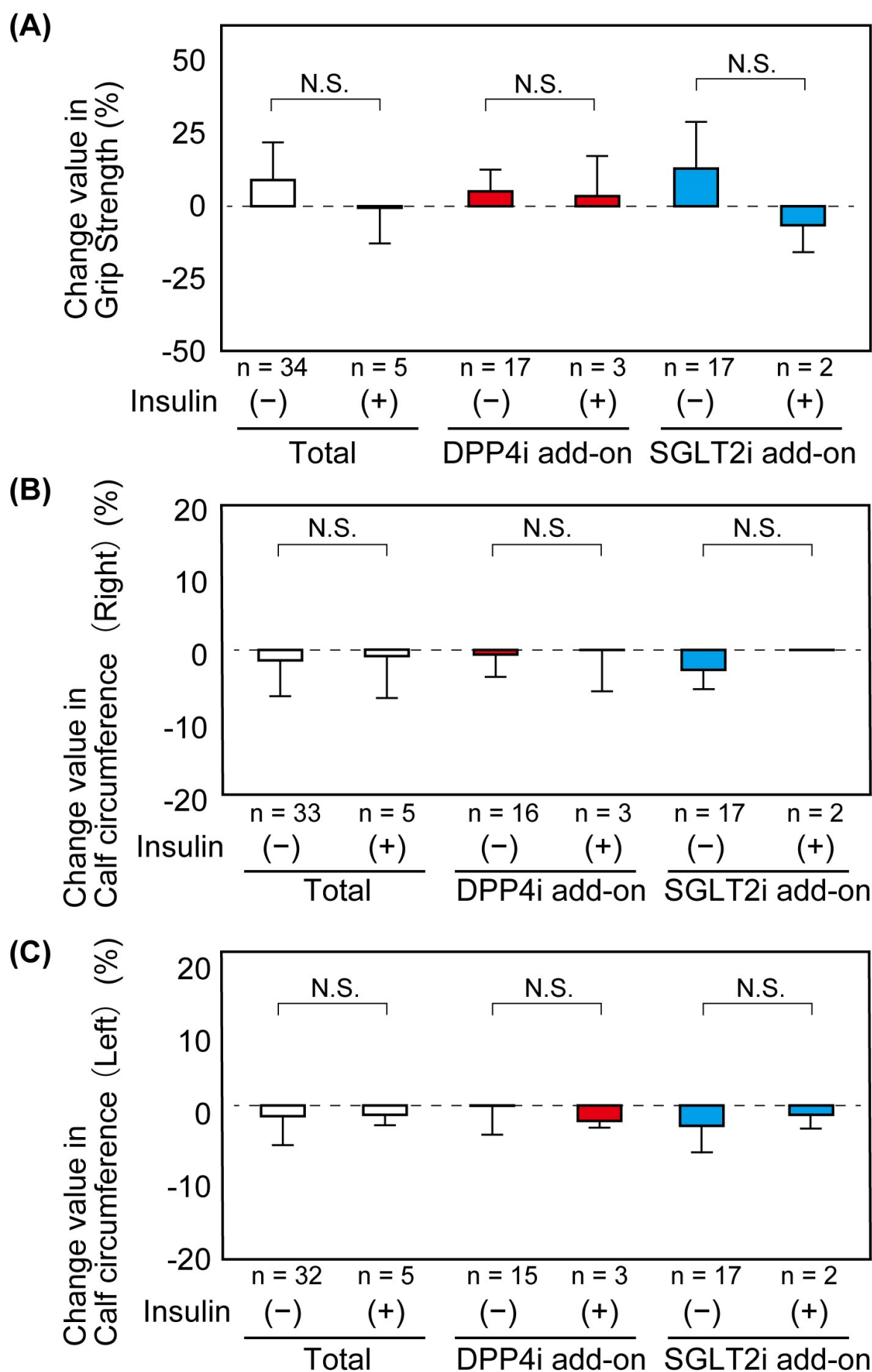

**Supplementary figure 2**

Supplement: Supplementary Figure 2 — Changes in grip strength and calf circumferences between patients with or without insulin administration Analyses were performed using Student’s t-test. DPP4i, dipeptidyl peptidase-4 inhibitor; SGLT2i, sodium-glucose cotransporter-2 inhibitor [file 2433-3298-7-3-0387-s002.pdf]
